# Postpartum Ovarian Vein Thrombophlebitis with Staphylococcal Bacteremia

**DOI:** 10.1155/2015/589436

**Published:** 2015-06-28

**Authors:** Eduardo Parino, Eric Mulinaris, Edgardo Saccomano, Juan Cruz Gallo, Gabriel Kohan

**Affiliations:** ^1^Department of Internal Medicine, Clínica y Maternidad Suizo Argentina, Avenida Pueyrredón 1461, C1118AAE Buenos Aires, Argentina; ^2^Department of Radiology, Clínica y Maternidad Suizo Argentina, Avenida Pueyrredón 1461, C1118AAE Buenos Aires, Argentina

## Abstract

A 34-year-old female patient presented with fever and right flank pain ten days after uncomplicated vaginal delivery. CT examination revealed right ovarian vein thrombosis and methicillin-resistant *Staphylococcus aureus* (MRSA) was isolated from blood cultures. No other source of bacteremia was found. Antibiotic therapy and anticoagulation with enoxaparin were instituted. Fourteen days after admission, she was discharged in good condition. Although a very uncommon complication after spontaneous vaginal delivery, septic ovarian vein thrombophlebitis should be suspected in cases of persistent puerperal fever when other diagnostic possibilities have been excluded.

## 1. Introduction

Septic ovarian vein thrombophlebitis is a rare complication during puerperium. Since its first description in the late 1800s, its incidence has fallen dramatically occurring in 1 in 3000 deliveries [1, 2]. It is more common after cesarean section (1/800) than after vaginal delivery (1/9000). In fact, more than 80% of cases have been described in women undergoing cesarean section mainly because of the higher rate of puerperal infection in this setting—a risk factor reported in 45 to 67% of cases in this entity [3]. Although the mortality rate has been largely reduced in the last decades [2], it can result in life-threatening conditions as sepsis, inferior vena cava thrombosis, and pulmonary embolism.

We report a case of a young woman who underwent uncomplicated vaginal delivery and was readmitted ten days later with fever and right flank tenderness. Septic ovarian vein thrombophlebitis was diagnosed associated with* Staphylococcus aureus* bacteremia.

## 2. Case Report

A 34-year-old woman, gravida 2 and parity 1, underwent spontaneous vaginal delivery after uneventful pregnancy. She did not require either uterine or placental instrumentation. Ten days later, she was readmitted to our facility with fever spikes (up to a maximum of 39.7°C) and right flank pain. Clinical examination only revealed right lower abdominal quadrant light tenderness. Uterine palpation did not elicit pain. Lochia were scarce and nonfetid. Laboratory at admittance only showed mild anemia (see Table 1 for more details). Urinalysis did not reveal pyuria.

Abdominal and gynecological ultrasound was also unrevealing.

Two sets of blood culture (two samples each) and urine culture were taken and the patient was empirically started on ceftriaxone 2 grs IV daily.

**Figure 1:**
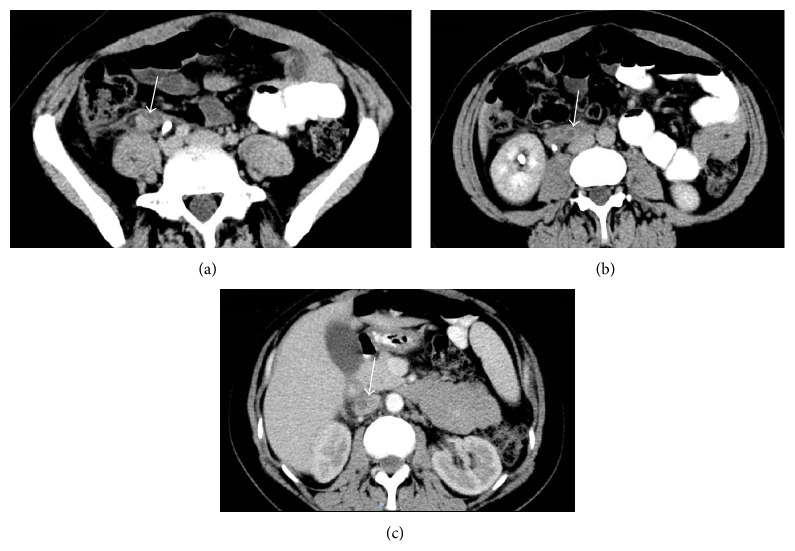
Axial contrast-enhanced CT images demonstrate enlargement of right gonadal (ovarian) vein with central hypodensity and enhancement of vessel wall (a-b) with cephalad extension to inferior vena cava (c). Inflammatory changes in adjacent retroperitoneal fat can also be seen.

A contrast-enhanced abdominal CT was performed which showed images suggesting the presence of thrombus in the right ovarian vein with extension to inferior vena cava (Figure 1). Doppler ultrasound confirmed these findings as no flow was detected. Enoxaparin 80 mg bid subcutaneously was initiated. Ceftriaxone was switched to vancomycin 1 gr IV bid when all samples of blood cultures yielded methicillin-resistant* Staphylococcus aureus*. Rifampin 300 mg bid orally was added. Urine culture was negative. On day 4 after admission, she became afebrile. Vancomycin serum concentration was monitored during hospitalization and dosing adjusted to achieve trough concentrations of at least 15 ug/mL.

Pulmonary embolism was ruled out by chest CT examination. Doppler ultrasound excluded deep vein thrombosis of the lower limbs.

A transesophageal echocardiogram did not show any valve vegetation. No other source of bacteremia was found.

She did not experience any other intercurrence until her discharge and after fourteen days of treatment with vancomycin, trimetoprim-sulfametoxazol (160/800) bid orally was indicated, according to pathogen susceptibility, for a total duration of antibiotic therapy of 3 weeks. Enoxaparin was switched to oral anticoagulation. Upon discharge, no adverse event was reported during follow-up.

The patient did not have a previous personal or family history for thrombosis and no other risk factor could be detected. Her obstetric history only recorded a spontaneous delivery at term of an uncomplicated pregnancy three years before.

## 3. Discussion

Two distinct forms of septic pelvic thrombophlebitis have been described: ovarian vein thrombophlebitis and deep septic pelvic thrombophlebitis. They have the same pathogenesis, may coexist, and probably represent the same process in different locations.

Even though this uncommon disorder has been associated with puerperal state, it has also been described with other conditions: pelvic inflammatory disease, pelvic surgery, and malignancy.

During postpartum period, venous stasis and hypercoagulability, in addition to endothelial damage caused by uterine infection, can be identified as the most important factors in the pathophysiology [4]. About 90% of reported cases are unilateral and right sided. Some authors proposed a retrograde flow in left ovarian vein during late pregnancy which would protect it against ascending infection [5].

Most patients develop spiking fever followed by right flank or lower abdominal tenderness during the first week after delivery. Ultrasound with Doppler examination can reveal the location and extent of the thrombus; however, the presence of overlying bowel gas may limit its usefulness. Contrast-enhanced CT and MRI angiography have a high sensitivity and specificity (100% versus 92% and 99% versus 100%, resp.) and are the preferred diagnostic tools [6].

Blood cultures provide evidence of a microorganism in less than 35% of the cases in earliest report [7]. More recent but small series have found a much lower rate of 3% [8]. Streptococci,* E. coli*, and anaerobes were the most common pathogens isolated.

Mortality in septic pelvic thrombophlebitis is very low (2%) but may be increased if septic emboli or systemic infection occurs [2].

Until the late sixties, the ligation of inferior vena cava was the treatment of choice. Since then, medical therapy with antibiotics and anticoagulants has gained consensus. With CT evidence of ovarian vein thrombosis, or involvement of iliac vein and vena cava, anticoagulation should be continued for 3 months or until resolution of thrombus in subsequent imaging. In case of pulmonary emboli vena cava filter placement should be considered. Length of antibiotic therapy is more controversial; some advocate a short course and discontinuation after clinical and leukocytosis improvement with at least 48–72 hours of apirexia, others recommend to maintain antimicrobials until hospital discharge; in the presence of septic emboli or positive blood cultures duration of treatment should be determined on an individual basis.

Septic ovarian vein thrombophlebitis with positive blood cultures, as in the case we report, seldom follows spontaneous vaginal delivery especially in the absence of risk factors as instrumentation or uterine manipulation, puerperal endometritis, or infection of episiotomy [8, 9], and methicillin-resistant* Staphylococcus aureus* (MRSA) is not the most prevalent pathogen that could be expected in this setting, as it has been stated above. MRSA is a challenging and emerging major problem in the healthcare system. In 2007, a reported case of MRSA sepsis complicated with necrotizing pneumonia arising from an infected episiotomy site illustrates the growing concern on the role of* Staphylococcus aureus* [10].

Clindamycin and gentamycin have been suggested as a good option of initial antibiotic therapy, probably because this combination is effective against bacteria that cause endometritis [11]. In 1988, Walmer et al. recommended the addition of ampicillin in order to provide broad coverage against enterococcal infections. Some strains of MRSA are susceptible to clindamycin but many are resistant. If its association with septic pelvic thrombophlebitis is reported more consistently in the future, vancomycin may be needed to be considered as part of the empiric regimen. Furthermore, we emphasize this approach in the setting of surgical site infection or postcesarean pelvic abscess as underlying conditions.

In summary, thrombophlebitis of ovarian vein is a difficult diagnosis that must be presumed in the setting of unexplained persistent fever during the first week of puerperium. In view of the rising rate of births by cesarean section, we might face this entity with increasing frequency in the years to come. Although much less common following uneventful vaginal delivery, it may occur, and despite its rarity, in the absence of an alternative diagnosis, it should not be excluded without an accurate assessment.

## Figures and Tables

**Table 1:** 

	Day 1	Day 4	Day 9	Before discharge
Hematocrit (%)	31	29	30	32
Hemoglobin (gr/dL)	10,4	9,9	10,0	10,6
White cell count (K/*μ*L)	9,62	4,54	5,9	4,15
Platelets (K/*μ*L)	147	—	313	—
Glucose (mg/dL)	130	93	90	86
Urea (mg/dL)	28	9	15	11
Creatinine (mg/dL)	0,8	0,6	0,7	0,7
Plasmatic sodium (mEq/L)	130	138	138	139
Plasmatic potassium (mEq/L)	4	3,7	4,2	3,5
ALT (UI/L)	16	—	—	—
AST (UI/L)	10	—	—	—
LDH (UI/L)	279	—	—	—
Prothrombin time (%)	70	—	29	38
APTT seg.	42	—	50	48
INR	—	—	2,76	2,16
VSG (mm/h)	—	55	—	—
CPR (mg%)	—	10,3	—	—
Vancomycin serum concentration (*μ*g/mL)	—	6	15	—
